# Genomic subtypes of breast cancer identified by array-comparative genomic hybridization display distinct molecular and clinical characteristics

**DOI:** 10.1186/bcr2596

**Published:** 2010-06-24

**Authors:** Göran Jönsson, Johan Staaf, Johan Vallon-Christersson, Markus Ringnér, Karolina Holm, Cecilia Hegardt, Haukur Gunnarsson, Rainer Fagerholm, Carina Strand, Bjarni A Agnarsson, Outi Kilpivaara, Lena Luts, Päivi Heikkilä, Kristiina Aittomäki, Carl Blomqvist, Niklas Loman, Per Malmström, Håkan Olsson, Oskar Th Johannsson, Adalgeir Arason, Heli Nevanlinna, Rosa B Barkardottir, Åke Borg

**Affiliations:** 1 Department of Oncology, Clinical Sciences, Lund University and Skåne University Hospital, Barngatan 2B, SE 22185 Lund, Sweden; 2 CREATE Health Strategic Center for Translational Cancer Research, Lund University, BMC C13, SE 22184, Lund, Sweden; 3 Lund Strategic Research Center for Stem Cell Biology and Cell Therapy, Lund University, BMC B10, SE 22184, Lund, Sweden; 4 Department of Pathology, Landspitali-University Hospital, 101 Reykjavik, Iceland; 5 Departments of Obstetrics and Gynaecology, Helsinki University Central Hospital, Helsinki, Finland; 6 Department of Pathology, Clinical Sciences, Lund University and Skåne University Hospital, SE 22185 Lund, Sweden; 7 Department of Pathology, Helsinki University Central Hospital, Helsinki, Finland; 8 Department of Clinical Genetics, Helsinki University Central Hospital, Helsinki, Finland; 9 Department of Oncology, Helsinki University Central Hospital, Helsinki, Finland; 10 Department of Oncology, Landspitali-University Hospital, 101 Reykjavik, Iceland; 11 Faculty of Medicine, University of Iceland, 101 Reykjavik, Iceland

## Abstract

**Introduction:**

Breast cancer is a profoundly heterogeneous disease with respect to biologic and clinical behavior. Gene-expression profiling has been used to dissect this complexity and to stratify tumors into intrinsic gene-expression subtypes, associated with distinct biology, patient outcome, and genomic alterations. Additionally, breast tumors occurring in individuals with germline *BRCA1 *or *BRCA2 *mutations typically fall into distinct subtypes.

**Methods:**

We applied global DNA copy number and gene-expression profiling in 359 breast tumors. All tumors were classified according to intrinsic gene-expression subtypes and included cases from genetically predisposed women. The Genomic Identification of Significant Targets in Cancer (GISTIC) algorithm was used to identify significant DNA copy-number aberrations and genomic subgroups of breast cancer.

**Results:**

We identified 31 genomic regions that were highly amplified in > 1% of the 359 breast tumors. Several amplicons were found to co-occur, the 8p12 and 11q13.3 regions being the most frequent combination besides amplicons on the same chromosomal arm. Unsupervised hierarchical clustering with 133 significant GISTIC regions revealed six genomic subtypes, termed 17q12, basal-complex, luminal-simple, luminal-complex, amplifier, and mixed subtypes. Four of them had striking similarity to intrinsic gene-expression subtypes and showed associations to conventional tumor biomarkers and clinical outcome. However, luminal A-classified tumors were distributed in two main genomic subtypes, luminal-simple and luminal-complex, the former group having a better prognosis, whereas the latter group included also luminal B and the majority of *BRCA2*-mutated tumors. The basal-complex subtype displayed extensive genomic homogeneity and harbored the majority of *BRCA1*-mutated tumors. The 17q12 subtype comprised mostly *HER2*-amplified and *HER2*-enriched subtype tumors and had the worst prognosis. The amplifier and mixed subtypes contained tumors from all gene-expression subtypes, the former being enriched for 8p12-amplified cases, whereas the mixed subtype included many tumors with predominantly DNA copy-number losses and poor prognosis.

**Conclusions:**

Global DNA copy-number analysis integrated with gene-expression data can be used to dissect the complexity of breast cancer. This revealed six genomic subtypes with different clinical behavior and a striking concordance to the intrinsic subtypes. These genomic subtypes may prove useful for understanding the mechanisms of tumor development and for prognostic and treatment prediction purposes.

## Introduction

The accumulation of genomic aberrations is a fundamental part of solid-tumor development. Identification of patterns of DNA copy-number alterations (CNAs) and the genes that are targeted may reveal underlying mechanisms of disease evolution and potential candidates for therapeutic intervention. Breast cancer (BC) is a profoundly heterogeneous disease that encompasses several distinct disease entities, in which correct stratification of patients is critical for optimal disease management. Conventional markers for prognostication or treatment prediction or both include tumor size and lymph-node involvement, histologic grade, estrogen (ER) and progesterone receptor (PgR) expression, as well as human epidermal growth factor receptor 2 (*HER2*, *HER2/neu*, *ERBB2*) amplification status. Recent development of high-throughput molecular methods offers new opportunities to capture the wide range of genomic and biologic variability in tumors. A pioneering study by Perou *et al. *[1] used a gene-expression signature to disclose five intrinsic molecular subtypes of BC: basal-like, normal-like, HER2-enriched, and luminal A and B, suggested to reflect differences in cellular origin and divergent progression of tumors. Subsequent analysis confirmed the relevance of these molecular subtypes by showing correlations to clinical parameters and overall survival (OS) [2].

A study of BC pathophysiology approached the subject of BC heterogeneity by linking transcriptional and genomic profiles [3]. By classifying tumors according to their patterns of CNA, three groups were identified and referred to as the 1q/16q, amplifier, and complex subtypes. Tumors of the 1q/16q genomic subtype showed better disease-specific survival as compared with the amplifier and complex subtypes, whereas tumors with a basal-like gene-expression profile were found predominantly within the complex genomic subtype [3].

In the present study, we analyzed high-resolution genomic data from array-comparative genomic hybridization (aCGH) analysis of 359 BCs to gain further insight into patterns of CNA and loci that are specifically targeted by focal amplification or homozygous deletion. We combined these results with gene-expression data to reveal underlying mechanisms of disease evolution to highlight genomic heterogeneity in BC. Hierarchical clustering analysis of significant genomic lesions revealed genomic subtypes that displayed different clinical outcomes and high similarity to the intrinsic gene-expression subtypes previously described [4]. Additionally, tumors from *BRCA1 *and *BRCA2 *mutation carriers were found in distinct genomic subtypes. In conclusion, we show that the genomic landscape of BC reveals subtypes that reflect biologic and clinical behavior.

## Materials and methods

### Patients and tumor material

Freshly frozen breast-tumor tissues (*n* = 359) were obtained from the Southern Sweden Breast Cancer Group tissue bank at the Department of Oncology, Skåne University Hospital, Lund, The Helsinki University Central Hospital, and Landspitali University Hospital. Of the 359 tumors, 346 were primary tumors, and the remaining cases were either local recurrences or lymph node metastases. Median OS follow-up time for patients from whom primary tumors were available was 8.1 years (range, 0.24 to 32 years). Tumor and patient characteristics are summarized in Table 1. Patients were diagnosed over a longer time period at different institutions and consequently not uniformly treated. The study was approved by the regional Ethical Committee in Lund (reg. no. LU240-01 and 2009/658), waiving the requirement for informed consent for the study, the Icelandic Data by the Protection Committee and the National Bioethics Committee of Iceland, and the Finnish Data by the Helsinki University Central Hospital Ethical Committee (207/E9/07). For Icelandic and Finnish patients, written informed consent was obtained according to the national guidelines.

**Table 1:** Patient and tumor characteristics for the 359 tumors

Number of tumors	359
Number of primary tumors	346
Number of primary *BRCA1*-mutated tumors	17
Number of primary *BRCA2*-mutated tumors	31
Number of primary familial non-*BRCA1/2 *tumors	126
Tumor size	
≤20 mm	145 (45%)
> 20 mm	175 (55%)
Mean size mm (SD)	25.8 (15)
Histologic grading	
Grade 1	26 (11%)
Grade 2	100 (43%)
Grade 3	106 (46%)
Estrogen-receptor status	
Positive	227 (66%)
Negative	119 (34%)
Progesterone-receptor status	
Positive	194 (57%)
Negative	149 (43%)
Lymph-node status	
Negative	190 (58%)
Positive	137 (42%)
Age	
Median age, years (range)	49 (27-88)
< 50 years	182 (51%)
≥ 50 years	174 (49%)
Classification according to Hu *et al. *[4]	
Basal-like	79 (22%)
Normal-like	34 (9%)
HER2-enriched	34 (9%)
Luminal A	95 (26%)
Luminal B	70 (19%)
Unclassified	47 (13%)
Overall survival	
Median overall survival in years (range)	8.1 (0.24-32)
Median overall survival in years for patients still alive (range)	12.8 (1.55-20.2)

Survival is given for the 346 patients from whom primary tumors were available.

### Gene-expression analysis

Global gene-expression analysis of breast tumors was performed by using oligonucleotide microarrays (Gene Expression Omnibus, GEO, [5] platform GPL5345) produced at the SCIBLU Genomics Centre at Lund University, Sweden [6], as described [7]. Data analysis and normalization [8] of the 359 tumors were performed, together with 218 other breast samples, as described (Additional File 1). Tumors were classified according to the intrinsic molecular subtypes reported by Hu *et al. *[4], a proliferation gene module [9], and a genomic-grade signature [10], as described [11] (Additional File 1). Gene-expression data for all 359 tumors are available through GEO as GSE22133.

### aCGH analysis

BAC microarrays (GEO platform GPL4723) comprising approximately 32,000 clones were produced by the SCIBLU Genomics Centre, Lund University, Sweden, as described [7]. Labeling, hybridization, image analysis, and initial data analysis were performed as described [7]. Technical replicate experiments were performed on 15 tumors. Copy-number estimates (log_2 _ratios) for each array were normalized [12], and replicated samples were merged after normalization. Breakpoint analysis was performed by using circular binary segmentation (CBS) with α = 0.01 [13]. Only segments of four or more BAC probes were used in further analyses. Gains and losses were detected by applying sample adaptive thresholds, derived from 250-kbp smoothed data, to CBS log_2 _ratios, as described [12]. Recurrent high-level amplifications were defined as occurring in > 1% of tumors with a CBS log_2 _ratio ≥1. The fraction of the genome altered (FGA) was calculated as described [14]. CGH data for all 359 tumors are available through GEO as GSE22133.

### Identification of significant CNAs by using GISTIC

Genomic Identification of Significant Targets in Cancer (GISTIC) [15] was used to identify significant amplification and deletion peaks in the 359 tumors, as described (Additional File 1). Student's *t *test performed on average scaled log_2 _ratios for significant GISTIC regions were used to identify regions associated with different clinical variables or molecular subtypes. Hierarchical clustering of significant GISTIC peaks was performed by using Pearson correlation and complete linkage on average scaled log_2 _ratios for each peak. Genomic coordinates for GISTIC regions are mapped to the UCSC Human Genome browser build 17 [16].

### Construction of gene-expression centroids based on genomic subtypes

Gene-expression centroids for genomic subtypes were created based on genes used for classification according to the molecular subtypes by Hu *et al. *[4] (Additional File 1). The centroids were subsequently applied to a previously reported [14] combined BC data set (*n* = 1,881 tumors) comprising 11 public BC data sets generated on Affymetrix U133A arrays, including the Chin *et al. *data set [3] (Additional File 1). Complementary aCGH data for the Chin *et al. *data set were processed and analyzed as described [17].

### Correlation of gene-expression data with genomic aberrations

Gene-expression data were compared with GISTIC aCGH log_2 _ratios for genomic subtypes by using Pearson correlation, as described [7]. GISTIC regions were expanded by one BAC probe in each direction to include borderline genes. A correlation cut-off representing a *P *value = 0.05 obtained from 100 permutations of aCGH sample labels was used to identify significantly correlated genes in GISTIC regions. Global correlation analysis for genomic subtypes by using genes mapped to individual BAC probes was performed similarly, with one modification; CBS log_2 _ratios were used for individual BAC probes.

### Survival analysis

Univariate and multivariate regression analyses of overall survival (OS) and distant metastasis-free survival (DMFS) were performed in R [18] by using the Survival package. OS or DMFS was the end point. Survival curves were compared by using Kaplan-Meier estimates and the log-rank test. The full follow-up time was used for log-rank tests and regression analyses, if not otherwise specified. Tick marks in Kaplan-Meier plots indicate censored follow-up.

## Results

### Comprehensive DNA copy-number analysis of BC

**Figure 1:**
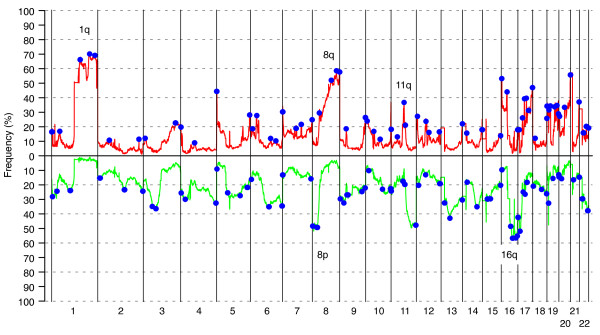
**Copy-number alterations (CNAs) observed in 359 breast cancers**. Blue regions indicate positions of significant genomic aberrations (*n* = 133) identified by Genomic Identification of Significant Targets in Cancer (GISTIC) analysis. Green corresponds to loss, and red, to gain. Most common CNAs are observed on chromosomes 1q, 8p, 8q, 11q, and 16q, as indicated in the figure.

We used a tiling BAC array CGH to survey genome-wide CNAs in BC from *BRCA1 *(*n* = 22) and *BRCA2 *mutation carriers (*n* = 32), non-BRCA1/2 familial (*n* = 132), and sporadic cases (*n* = 173). The overall pattern of DNA CNAs displayed an extensive heterogeneity with most frequent CNAs on chromosomes 1q, 8q, and 16q (Figure 1). The GISTIC algorithm [15] was used to identify genomic changes that represented statistically significant events by using all 359 tumors. GISTIC identified 66 regions of gain and 67 regions of loss (Figure 1, Additional File 2).

**Figure 2:**
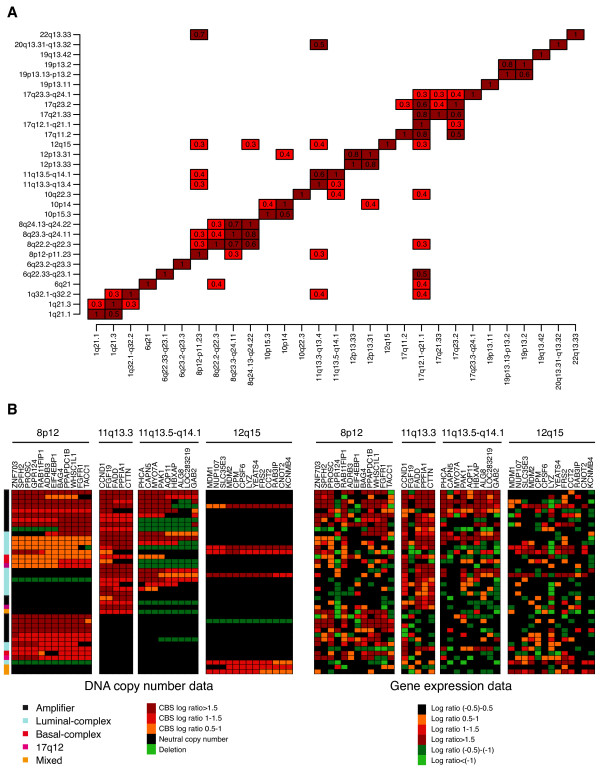
**High-level amplifications in breast cancer (BC)**. **(a) **Coamplification patterns in BC. For each amplification (vertical axis), the fraction of samples with a coamplification (horizontal axis) is indicated in each box. Coamplification fractions smaller than 20% are excluded: for example, 30% of all 12q15-amplified samples also have 8p12 amplification, whereas the fraction of 8p12-amplified samples with 12q15 amplification is < 0.2 and is not displayed. **(b) **Overview of the coamplification pattern in chromosomes 8p12, 11q13, and 12q15. Amplification pattern is also evident on a gene-expression level, where a number of genes show a significant relation to gene-dosage effects.

The most frequent high-level amplification peaks were found on chromosomes 17q12 (13%), 8p12 (7%), 8q24.21 (6%), 11q13.3 (6%), and 11q13.5 (4%), encompassing known oncogenes such as *HER2*, *MYC*, *CCND1*, and *PAK1 *(Table 1 in Additional File 3). To identify coamplified regions, we selected loci amplified in at least three cases and determined the fraction of coamplified samples (Figure 2a). Amplifications located on the same chromosome or chromosomal arm were more commonly coamplified; however, chromosomes 11q13 and 8p12 were also coamplified in a significant fraction (Figure 2a). Additionally, chromosome 12q15 was found to be coamplified with 8p12 and 11q13. Several other loci were coamplified but contained too few cases to draw any reliable conclusions. As expected, the coamplification pattern also was evident on the gene-expression level, pinpointing novel and known target genes (Figure 2b).

In a subsequent step we aimed to identify target genes within high-level amplifications. In total, we found 31 loci affected by high-level amplifications. Integration of gene-expression data with DNA copy-number data revealed a number of candidate genes in which mRNA expression was significantly correlated with gene dosage. Among significantly correlated genes were known oncogenes, such as *HER2*, *FGFR1*, *CCND1*, *MYC*, and *MDM2*; however, we also found, for example, *MDM4*, *ELK4*, *PTPRK*, *GAB2*, and *RAB22A *to be significantly correlated (Table 2). Among the top significant deletion GISTIC peaks were chromosomes 9p21.3 and 10q23.31, encompassing the *CDKN2A *and *PTEN *genes, respectively (Additional File 2).

**Table 2:** Regions amplified with a frequency of >1% in the 359 tumors

Amplified region	Percentage of amplified samples	Significantly correlated genes
1q21.1	1.1	*BCL9, ACP6*
1q32.1-q32.2	1.9	*BTG2, ATP2B4, SOX13, GOLT1A, PLEKHA6, PPP1R15B, MDM4, RBBP5, RIPK5, ELK4, RAB7L1, SRGAP2, IKBKE, LGTN, DYRK3, MAPKAPK2*,
6q21	1.4	*BVES, AIM1*
6q22.33-q23.1	1.1	*PTPRK, L3MBTL3*
8p12-p11.23	5.8	*ZNF703, SPFH2, PROSC, RAB11FIP1, ADRB3, BAG4, PPAPDC1B, WHSC1L1, FGFR1*
8q22.2-q22.3	3.1	*VPS13B, RNF19, ANKRD6, GRHL2, EDD1, AZIN1, ATP6V1C1, FZD6, SLC25A32*
8q23.3-q24.11	6.1	*TRPS1, THRAP6, EXT1*
8q24.13-q24.22	6.7	*ZHX1, ATAD2, C8orf32, FBXO32, TMEM65, TRMT12, RNF139, TATDN1, KIAA0196, C8orf36, TRIB1, FAM84B, MYC, DDEF1 *
10p15.3	1.1	*PFKP, PITRM1*
10p14	1.4	
10q22.3	1.4	*POLR3A, RAI17, PPIF*
11q13.3-q13.4	6.4	*CCND1, FADD, PPFI1A, CTTN*
11q13.5-q14.1	3.6	*PHCA, CAPN5, MYO7A, PAK1, HBXAP, ALG8, LOC283219, GAB2*
12p13.33	1.7	*JARID1A, WNK1, RAB6IP2, FBXL14, WNT5B, ADIPOR2, DCP1B*
12p13.31	1.4	*CD9, TNFRSF1A, LTBR, TAPBPL, MRPL51, NOL1, CHD4, ING4, ZNF384, COPS7A, MLF2, PTMS, USP5, SPSB2, ENO2, ATN1, PTPN6, PHB2, C1 S, C1RL, CLSTN3, PEX5*
12q15	1.9	*MDM1, NUP107, SLC35E3, MDM2, CPSF6, YEATS4, FRS2, CCT2, CNOT2*
17q11.2	3.6	*UNC119, PIGS, ALDOC, SPAG5, KIAA0100, SDF2, SUPT6 H, RAB34, NEK8, ERAL1, FLOT2, PHF12, LOC116236, GIT1, ANKRD13B, CORO6, SSH2*
17q12.1-q21.1	13.6	*FBXL20, PPARBP, STARD3, PERLD1, HER2, C17orf37, GRB7, GSDML, ORMDL3, PSMD3, THRAP4 *
17q21.33	4.2	*LOC81558, ITGA3, PDK2, PPP1R9B, XYLT2, MRPL27, FLJ20920, RSAD1, EPN3, ABCC3, ANKRD40, TOB1*
17q23.2	3.1	*TRIM25, AKAP1, MSI2*
17q23.2	3.1	*TMEM49, TUBD1, ABC1, USP32, APPBP2, BCAS3*
17q23.3-q24.1	2.5	*CYB561, WDR68, MAP3K3, LYK5, DDX42, FTSJ3, PSMC5, SMARCD2, ERN1, POLG2, DDX5, SMURF2*
19p13.13-p13.2	1.1	*SIPA1L3, PPP1R14A, SPINT2, YIF1B, EIF3S12*
19p13.2	1.1	*FBL, PSMC4, FLJ36888, PLD3*
19q13.42	1.4	*SUV42OH2, KLP1, ZNF579, FLJ14768, ZNF524, LOC147808, ZNF580, ZNF581*
20q13.31-q13.32	1.1	*RAE1, RNPC1, RAB22A, VAPB, STX16, GNAS, TH1L, CTSZ*

Genes with significant correlation (*P *< 0.05) between mRNA expression and DNA copy-number status are indicated for each region.

### Molecular classification of BC by using DNA copy-number alterations

**Figure 3:**
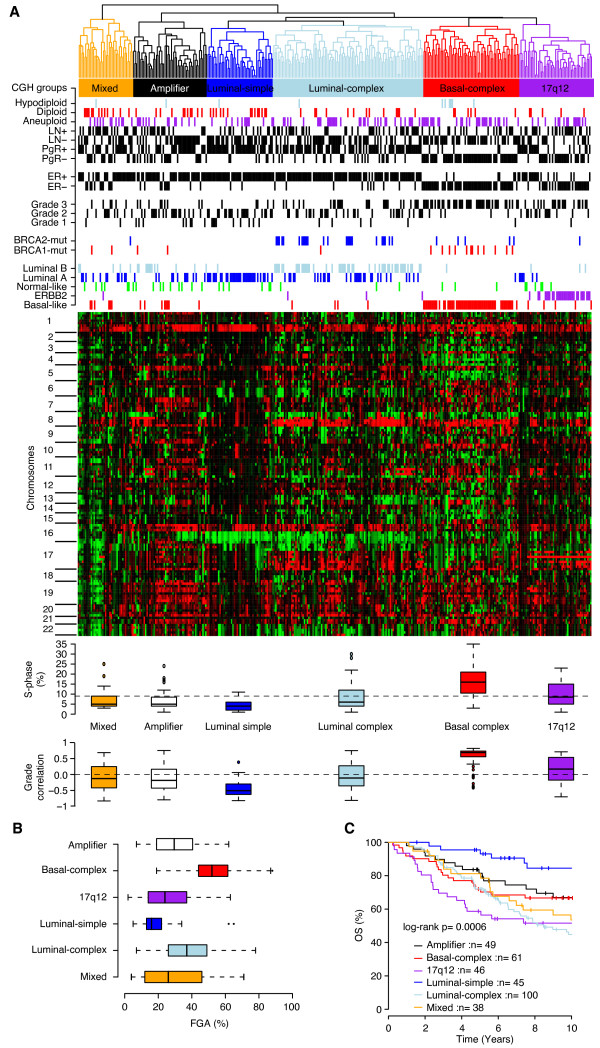
**Unsupervised analysis of Genomic Identification of Significant Targets in Cancer **(**GISTIC) regions identifies six CGH subgroups of breast cancer (BC) associated with different clinical and molecular characteristics**. **(a) **Hierarchic clustering of 133 GISTIC regions identifies six subtypes with different clinical and molecular characteristics, and genomic aberrations. Horizontal dashed line for S-phase indicates the average across all samples. **(b) **Fraction of the genome altered (FGA) for genomic subtypes indicating that basal-complex samples are genomically unstable, whereas luminal-simple tumors are genomically stable. **(c) **Overall survival (OS) for 339 patients, for whom primary tumors were available, classified according to genomic subtypes, mirrors results obtained for the intrinsic gene-expression subtypes.

BC has been divided into intrinsic subtypes by using gene-expression profiling [1,2]. Consistent with previous reports [3,19-21], we found several chromosomal regions that were differentially altered between the intrinsic gene-expression subtypes, as well as for several clinical parameters (Figures S1 to S3 in Additional File 3). We used GISTIC regions derived from DNA copy-number data and hierarchical clustering to divide our cohort into six subtypes characterized by different FGA levels and CNAs (Figure 3a, b; Table 3; Additional File 4; Figure S4 in Additional File 3). To further characterize the identified genomic subtypes, we used available classification according to the intrinsic gene-expression subtypes, as well as other signatures derived from gene-expression analysis of all tumors.

**Table 3:** Patient and tumor characteristics for the six genomic subtypes

	Basal-complex *n *(%)	17q12 *n *(%)	Luminal-complex *n *(%)	Luminal-simple *n *(%)	Amplifier *n *(%)	Mixed *n *(%)
Group size	67	51	105	46	52	38
Hereditary status						
*BRCA1*-mutated	17 (25)	0	1 (1)	0	2 (4)	2 (5)
*BRCA2*-mutated	5 (7)	1 (2)	25 (24)	0	0	1 (3)
Familial	15 (22)	19 (37)	38 (36)	23 (50)	29 (56)	8 (21)
Sporadic	30 (45)	31 (61)	41 (39)	23 (50)	21 (40)	27 (71)
Clinical parameters						
ER^+^	7 (10)	19 (37)	91 (87)	44 (96)	40 (77)	26 (68)
ER^-^	56 (84)	29 (57)	11 (10)	2 (4)	10 (19)	11 (29)
LN^+^	19 (28)	25 (49)	42 (40)	12 (26)	20 (38)	19 (50)
LN^-^	43 (64)	19 (37)	50 (48)	32 (70)	28 (54)	18 (47)
Mean tumor size (mm ± SD)	27 ± 12	31 ± 21	26 ± 17	21 ± 10	25 ± 13	25 ± 11
Histologic grade 1	1 (1)	1 (2)	5 (5)	9 (20)	8 (15)	2 (5)
Histologic grade 2	5 (7)	14 (27)	31 (30)	21 (46)	19 (37)	10 (26)
Histologic grade 3	42 (63)	19 (37)	26 (25)	4 (9)	9 (17)	6 (16)
Molecular subtypes by Hu *et al. *[4]						
Basal-like	56 (84)	5 (10)	4 (4)	0	8 (15)	6 (16)
HER2-enriched	0	30 (59)	3 (3)	0	0	1 (3)
Luminal B	1 (1)	3 (6)	48 (46)	1 (2)	12 (23)	5 (13)
Luminal A	1 (1)	6 (12)	31 (30)	33 (72)	12 (23)	12 (32)
Normal-like	3 (4)	6 (12)	3 (3)	6 (13)	10 (19)	6 (16)
Unclassified	6 (9)	1 (2)	16 (15)	6 (13)	10 (19)	8 (21)
FGA (mean %)						
Overall FGA (%)	53	26	37	19	31	29
Gain FGA (%)	18	12	15	9	15	12
Loss FGA (%)	34	14	22	10	16	18

ER, Estrogen receptor; FGA, fraction of the genome altered; LN, lymph node.

Four of the genomic subtypes displayed striking similarity to previously described intrinsic subtypes derived from gene-expression profiling. The 17q12 subtype comprised 67% of all cases with *HER2 *amplification (segmented log_2 _ratio > 0.5), and 88% of all HER2-enriched intrinsic subtype-classified cases were found in this genomic subgroup. The basal-complex subtype included mainly basal-like classified tumors, as well as 77% of all *BRCA1*-mutated tumors. Furthermore basal-complex tumors displayed higher S-phase fractions, higher FGA percentages, and higher correlation to a genomic-grade signature (Figure 3a). The luminal-simple subtype included mainly luminal A-classified tumors (72%) and was predominantly characterized by frequent losses on 16q, and gains on 1q and 16p. Furthermore, cases in this subtype displayed lower FGA levels, S-phase fractions, and genomic grade correlation. Finally, the luminal-complex subtype included 69% of all luminal B-classified cases, and 78% of all *BRCA2*-mutated tumors, but also 33% of all luminal A subtype-classified tumors. Moreover, two groups of tumors displayed mixed intrinsic subtype characteristic and heterogeneous genomic profiles (the amplifier and mixed subtypes). The mixed subtype showed heterogeneous losses across several chromosome arms, most frequently on 8p, 13q, and 17p, together with frequent gain on 1q (~60%) (Additional File 4). The amplifier subtype contained 48% of the 8p12-amplified cases (segmented log_2 _ratio > 0.5) as well as frequent gain of 1q, 11q13, 16p, 19p, and 19q and frequent losses on 8p, 11qter, 16q, and 17p (Additional File 4). Furthermore, the amplifier contained the highest fraction of luminal B-classified cases after the luminal-complex subtype. However, several GISTIC regions distinguished amplifier-classified cases from luminal-complex cases in a supervised analysis (Figure S5 in Additional File 3). Interestingly, 31% of all luminal-complex samples harbored 8p12-amplification; however, these tumors displayed differences in the genomic pattern compared with 8p12-amplified tumors in the amplifier subclass. First, 8p12-amplified tumors in the luminal-complex subtype showed higher FGA compared with 8p12-amplified tumors in the amplifier subtype (*P *= 0.02; *t *test). Second, in the amplifier subtype, 86% and 71% of 8p12-amplified tumors showed coamplification of the 11q13.5 and 11q13.3 GISTIC regions, respectively, as compared with 29% and 17% of 8p12-amplified tumors in the luminal-complex subtype.

To investigate whether the identified genomic subtypes showed an association with the outcome, we performed Kaplan-Meier analyses by using OS as end point. Not surprisingly, significant differences in OS between genomic subtypes were observed, with the luminal-simple subtype having the best outcome, and the 17q12 subtype, the worst outcome (Figure 3c).

### Association of BRCA1/2 mutation status and genomic subtypes

**Figure 4:**
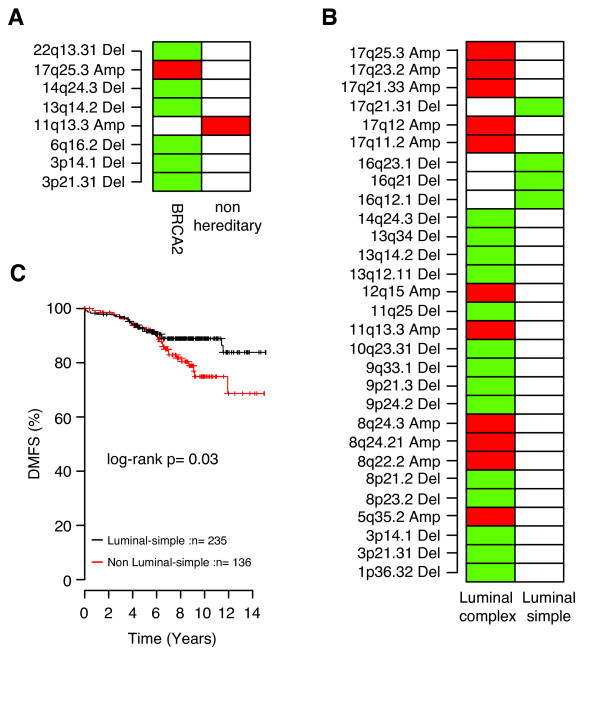
**Supervised analysis in luminal genomic subtypes**. **(a)** Significant Genomic Identification of Significant Targets in Cancer (GISTIC) regions between *BRCA2*-mutated and non-*BRCA2 *tumors within the luminal-complex subtype. **(b)** Significant GISTIC regions between the luminal-complex and luminal-simple subtypes. **(c) **Distant metastasis-free survival (DMFS) for luminal A tumors stratified by classification as luminal-simple or non-luminal-simple in a combined Affymetrix gene-expression data set. Significant GISTIC regions were identified by Bonferroni-adjusted Student *t *test (*P *< 0.05); red indicates more-frequent gain, and green indicates more-frequent loss, in comparisons between GISTIC regions. Only significant regions with ≥20% CNA frequency are displayed.

The basal-complex and the luminal-complex subtypes contained 77% of all *BRCA1- *and 78% of all *BRCA2*-mutated tumors, respectively. Interestingly, by using supervised analysis, no GISTIC region was found to differ significantly between *BRCA1 *and non-*BRCA1 *tumors in the basal-complex subtype, although the former showed a significantly higher FGA (*P *= 0.01; *t *test). The five *BRCA1*-mutated tumors falling outside the basal-complex subtype did not differ significantly from other *BRCA1 *tumors regarding FGA, S-phase fraction or genomic grade correlation, with the exception that two of five tumors were ER positive. *BRCA2 *tumors in the luminal-complex subtype were characterized by losses on 3p21.31, 3p14.1, 6q16.2, 13q14.2, 14q24.3, and 22q13.31 and gains on 17q25.3 compared with non-*BRCA2 *tumors in the same genomic subtype, whereas the latter showed more-frequent gain of 11q13.3 (Figure 4a). Again, *BRCA2 *tumors in the luminal-complex subtype showed significantly higher FGA than did non-*BRCA2 *tumors in this subtype (*P *= 0.01; *t *test). The seven *BRCA2*-mutated tumors not belonging to the luminal-complex subtype were found in the basal-complex (*n* = 5), 17q12 (*n* = 1), and mixed (*n* = 1) subtypes. *BRCA2 *tumors in the basal-complex subtype were ER-negative (80%), showed higher FGA, higher genomic grade correlation than luminal-complex *BRCA2 *tumors, and displayed CNAs more similar to *BRCA1 *tumors.

### DNA copy-number alterations divide luminal breast cancer in two entities

Supervised analyses were performed to investigate differences in characteristic alterations between luminal-complex and luminal-simple tumors. Luminal-simple tumors were primarily characterized by frequent loss on 16q, whereas luminal-complex tumors were characterized by losses on 3p, 8p, 9p, 11q25, and 13q and gains on 8q, 11q13.3, and 17q (Figure 4b). Interestingly, luminal A tumors in the luminal-simple subtype showed several distinct differences compared with luminal A tumors in the luminal-complex subtype, including lower FGA (*P *= 0.0005; *t *test), different CNA pattern (Figure S6 in Additional File 3), lower genomic-grade correlation (*P *= 0.02; *t *test), lower correlation to the luminal B gene-expression centroid [4] (*P *= 0.0005; *t *test), higher correlation to the luminal A gene-expression centroid [4] (*P *= 0.05; *t *test), and a trend toward better OS (log-rank, *P *= 0.06). By comparison, in the luminal-complex subtype, luminal A cases showed better OS than luminal B cases (log-rank, *P *= 0.02), as well as lower FGA, S-phase fractions, and genomic grade correlation (*P *< 0.02; *t *test).

To confirm the observation that luminal A cases in the luminal-simple subtype show a trend toward better clinical outcome than other luminal A cases, we created gene-expression centroids for the genomic subtypes based on genes from the Hu *et al. *[4] gene list (Additional File 1). Gene-expression centroids were subsequently applied to a previously reported combined Affymetrix BC data set [14]. For luminal A-classified tumors in the combined Affymetrix data set, improved DMFS was observed for luminal-simple samples compared with non-luminal-simple samples (Figure 4c), further supported by multivariate analysis (*n* = 225, *P *= 0.004; HR = 0.36; 95% CI = 0.18-0.73) using LN status, ER status, tumor size, and histologic grade (grade 3 versus 1 and 2) as covariates. To investigate whether luminal-simple-classified luminal A tumors also showed lower FGA in independent data sets, we analyzed the Chin *et al. *[3] aCGH data set by using the genomic-subtype classification from corresponding gene-expression data in the combined Affymetrix data set. Convincingly, for luminal A tumors in the Chin *et al. *[3] data set, luminal-simple cases showed lower FGA than did luminal-complex cases (*P *= 0.01; *t *test) and a trend towards better OS (log-rank, *P *= 0.16).

### High-level amplifications in genomic subtypes

High-level amplifications were observed in 41 of the 66 GISTIC gain regions, involving 139 (39%) of 359 tumors. Interestingly, none of these belonged to the luminal-simple subtype. Occurrence of high-level amplifications was associated with a worse OS (log-rank, *P *= 0.0007). The 17q12 subtype showed the highest percentage of tumors with high-level amplifications (78%), followed by the amplifier (50%), luminal-complex (38%), basal-complex (34%), and mixed (26%) subtypes. Certain high-level amplicons were predominantly observed in specific subtypes: for example, 10p14 (basal-complex), 17q11.2 (17q12 subtype), 17q12 (17q12 subtype), 17q21.33 (17q12 subtype), and 19p13.11 (17q12 subtype). Other amplicons were observed in two subtypes: for example, 8p12 (amplifier, luminal-complex), 11q13.3 and 11q13.5 (luminal-complex, amplifier), 12p13.31 (basal-complex, amplifier), 6q23.3 (basal-complex, mixed) and 8q24.21 (luminal-complex, basal-complex).

## Discussion

BC is a biologically heterogeneous disease and has been stratified into molecular subtypes by using gene-expression profiling [1]. These subtypes were subsequently found to display different clinical outcomes with the HER2-enriched, basal-like, and luminal B as poor prognostic groups [2]. Several studies have used microarray-based genome-wide DNA copy-number analysis to describe recurrent, almost universally affected, chromosomal regions on 1q, 8, and 16, but also recognized that BC is a profoundly heterogeneous disease on the genomic level [3,21-24].

Here, we used high-resolution BAC aCGH to confirm the frequent CNAs on chromosomes 1q, 8q, and 16, as well as recurrent high-level amplifications on 17q12, 11q13, 8p12, and 8q24, encompassing known oncogenes, as previously described [3]. Although these CNAs may have a major impact on the progression of a significant proportion of BC, other amplicons contribute to the genomic diversity of the disease. For instance, chromosome 1q32 was highly amplified in seven (2%) of the 359 tumors and includes the *MDM4 *gene. A significant correlation was found between gene and transcript levels, suggesting that *MDM4 *is a potential oncogene in BC with a p53-inhibiting function similar to that of *MDM2 *[25]. Coamplification of chromosomes 8p12 and 11q13.3, occasionally combined with 11q13.5-q14.1 or 12q15, was found in a significant fraction of tumors. This suggests that the targeted genes or oncogenic pathways act synergistically and are advantageous for the tumor: for example, *CCND1 *(11q13.3) targeting the G_1_/S checkpoint, and *MDM2 *(12q15) targeting the p53 pathway. Previous studies also indicated frequent coamplification of 11q13 and 8p12, in which functional analyses revealed an extended complexity of these two cooperating genetic events [26]. The GISTIC algorithm [13] also captured other rare high-level gene amplifications such as the *PIK3CA *and *MYB *loci, genes frequently affected by alternative mechanisms [27,28], supporting their role in BC development.

Unsupervised hierarchical clustering based on significant (GISTIC) CNAs categorized the cohort into six genomic subtypes with striking similarity to the previously described gene-expression subtypes and differences in clinical outcome [1]. Earlier studies recognized three genomic subtypes in BC, characterized by a 1q/16q complex and amplifier genotype, respectively [3,21]. Their complex subtype was associated with the basal-like gene-expression subtype [3], corroborating our observation of a genomically homogenous basal-complex subtype. The 1q/16q subtype most likely corresponds to our luminal-simple subtype, whereas we, because of a larger number of tumors, were able to divide the amplifier subtype [3,21] further into one homogenous 17q12 subtype comprising the majority of *HER2*-amplified cases, a luminal-complex, and an amplifier subtype. The 17q12 subtype was associated with the worst clinical outcome and was essentially unified by a few amplified regions on 17q, including the *HER2 *locus on 17q12, as discussed in more detail elsewhere [17]. Moreover, 8p12-amplified tumors were confined mainly to two genomic subtypes, the amplifier and luminal-complex subtypes, with high-level 8p12 amplification occurring primarily in the amplifier subtype. In agreement with the findings by Chin *et al. *[3], we found that tumors with gain or loss at the 8p12 GISTIC region displayed inferior survival as compared with tumors with normal 8p12 copy number (log-rank, *P *= 0.04 and *P *= 0.008, respectively).

As expected, 17 of 22 tumors from *BRCA1 *mutation carriers were located in the basal-complex subtype, confirming the complex genome of *BRCA1 *tumors [29,30] and previous gene-expression studies [2]. Interestingly, no significant CNA difference was found between *BRCA1 *and non-*BRCA1 *tumors in the basal-complex subtype, indicating an extensive genomic homogeneity in this genomic subtype. We did observe a significantly higher FGA in *BRCA1 *tumors, although it should be noted that all basal-complex tumors had significantly higher FGA than did non-basal-complex tumors. These results may point toward a general DNA-repair deficiency of tumors in the basal-complex subtype, which may extend recent therapeutic opportunities beyond patients with a germline *BRCA1 *mutation [31]. Basal-complex tumors had frequent copy-number losses on chromosome 5q, in line with previous studies showing loss of heterozygosity (LOH) and physical deletions of 5q in *BRCA1 *tumors [30,32]. Although a target gene in this region is still to be identified, several candidates do exist (for example, *PIK3R1 *located on chromosome 5q13.1, previously found to be homozygously deleted in a *BRCA1*-mutated tumor) [32].

The majority of *BRCA2*-mutated tumors were located in the luminal-complex subtype, the exception being a few predominantly basal-like and ER-negative *BRCA2 *tumors that were in the basal-complex subtype. Deletions on chromosomes 3p, 6q, and 13q14 and gains on 17q were more frequent in *BRCA2 *tumors as compared with other luminal-complex tumors, as also was shown by others [30,33], and is somewhat mirrored by the higher frequency of CNAs in *BRCA2 *tumors. Taken together, in contrast to the scenario for *BRCA1*, these results indicate that specific *BRCA2*-associated genomic aberrations exist.

In strong contrast, tumors of the luminal-simple genomic subtype displayed a stable genome without high-level amplifications and with CNAs primarily on chromosomes 1q and 16q. This subtype included almost exclusively ER-and PgR-positive tumors of low histologic grade, and included approximately one third of all luminal A-classified samples. However, another third of luminal A tumors were found in the luminal-complex subtype, and 13%, in the amplifier subtype, suggesting heterogeneity within the current gene-expression-based classification of luminal A tumors. Luminal A tumors in the luminal-complex subtype were characterized by a significantly higher FGA and higher correlation to the genomic-grade signature [10], as well as a different pattern of genomic alterations than luminal A tumors of the luminal-simple group. Most important, the latter cases showed a trend toward better survival, supporting the aim of a clinically meaningful stratification of luminal A tumors based on their pattern of DNA CNAs.

To test this hypothesis, we constructed gene-expression centroids for the different genomic subtypes and applied them to two independent breast cancer gene-expression data sets. Intriguingly, an improved clinical outcome was observed for luminal-simple-classified samples within the luminal A subtype in a large combined Affymetrix data set, as well as lower FGA in the Chin *et al. *[3] data set. Corroborating findings by Chin *et al.*, this strongly suggests that the luminal A subtype could be further divided based on genomic alterations, warranting further investigation. Previously, reports suggested that a fraction of histologic grade 3 tumors progressed from grade 1 with accumulation of genomic aberrations [34,35]. An interesting hypothesis would be that the luminal-simple and luminal-complex division reflects a tumor-progression pathway of luminal tumors, as the frequent genomic aberrations in luminal-simple cases (+1q, -16q) are also apparent in luminal-complex samples. Moreover, luminal-complex tumors have additional genomic aberrations not present in luminal-simple tumors, such as +8q, -11q, and -13q, suggesting that these represent late genomic events. However, more in-depth studies are needed to confirm this.

## Conclusions

Six main groups of BC with distinct genomic-aberration patterns and striking similarity to gene-expression subtypes were found. *BRCA1 *tumors were confined to a uniform subtype termed basal-complex, characterized by a high frequency of low-level CNAs, basal-like, ER and PgR-negative, and histologic grade 3 tumors. *BRCA2 *tumors clustered among luminal-complex tumors characterized mostly as luminal B, ER-positive, and histologic grade 2. The genomic subtypes were significantly associated with clinical outcome, and the observation that luminal-simple cases display a better disease outcome within the intrinsic luminal A subtype was validated in independent data sets. Finally, our data emphasize the profound molecular heterogeneity in BC. Understanding the underlying genomic and biologic mechanisms may prove useful for prognostic as well as treatment-prediction purposes.

## Abbreviations

aCGH: Array-comparative genomic hybridization; BC: breast cancer; CBS: circular binary segmentation; DMFS: distant metastasis-free survival; ER: estrogen receptor; FGA: fraction of the genome altered; GEO: gene-expression omnibus; GISTIC: genomic identification of significant targets in cancer; LN: lymph node; LOH: loss of heterozygosity; OS: overall survival; PgR: progesterone receptor.

## Competing interests

The authors declare that they have no competing interests.

## Authors' contributions

GJ, JS, and ÅB conceived of the study. JS, GJ, AA, JVC, KH, CH, OK, RF, CS, and HG performed array experiments. JS performed data analysis with support by MR, JVC, and GJ. GJ and JS wrote the manuscript with the assistance of MR, JVC, and ÅB. BA, NL, LL, OJ, KA, PH, CB, HO, PM, RB, and HN contributed samples and clinical information. All authors approved the final manuscript.

## Supplementary Material

Additional file 1 **A pdf document containing supplementary information about methods used and data processing**. Click here for file

Additional file 2 **An Excel table listing the 133 significant GISTIC regions**. Click here for file

Additional file 3 **A pdf file containing one supporting table and six supporting figures**. The supporting table describes recurrent high-level amplifications found in the 359 tumors. Supporting Figure 1 describes differences in CNAs and FGA associated with clinical variables in the 359 tumors. Supporting Figure 2 describes differences in CNAs, FGA, and outcome associated with the intrinsic gene-expression subtypes in the 359 tumors. Supporting Figure 3 shows CNA frequency for the intrinsic gene-expression subtypes in the 359 tumors. Supporting Figure 4 describes CNAs associated with the genomic subtypes. Supporting Figure 5 describes differences in CNAs between the luminal-complex and amplifier genomic subtypes. Supporting Figure 6 describes differences and frequencies of CNAs between luminal A tumors classified as luminal-simple or luminal-complex, as well as luminal B tumors classified as luminal-complex. Click here for file

Additional file 4 **A pdf file showing CNA frequency in the genomic subtypes**. Click here for file
