# Supplementary material for: Genomic subtypes of breast cancer identified by array-comparative genomic hybridization display distinct molecular and clinical characteristics
Source: Breast Cancer Res. 2010 Jun 24;12(3):R42. doi: 10.1186/bcr2596 (PMC2917037; doi:10.1186/bcr2596)
Supplement: Additional file 1 — A pdf document containing supplementary information about methods used and data processing. [file bcr2596-S1.pdf]

Supplementary Material and Methods

Identification of significant copy number alterations using GISTIC

The GISTIC [15] implementation applied for detection of significant copy number alterations used a fixed log_2ratio threshold of ± 0.1 for calculation of the G-score. Consequently, prior to GISTIC analysis segmented CBS log_2ratios for each sample were rescaled so that an individual sample's sample adaptive threshold (Additional File 2) was set to ± 0.1. For each tumor, gain then corresponds to a GISTIC log_2ratio>0.1 and loss to < -0.1 in rescaled log_2ratio. CNV masking was performed by matching BAC probes to CNV data for the NCBI build 35 using the Toronto Database of Genomic Variants. GISTIC regions were defined as GISTIC wide-peak limits and mapped to the hg17 build [16]. Only GISTIC peaks with a q-value <0.01 were used in subsequent analysis (n=133). Student's t-tests on average log_2ratios for GISTIC regions were used to identify regions associated with different clinical variables. A Bonferroni-adjusted p-value <0.05 was considered significant. Analysis was performed using the R-package multtest [18]. Genes in GISTIC regions were identified by matching GISTIC coordinates to the hg17 RefGene file obtained from dChip website (Cheng Li Lab).

Gene expression analysis

The 359 tumors were processed together with 218 other breast cancers as described (Vallon-Christersson et al. manuscript in preparation). Hybridizations were performed on Gene Expression Omnibus (GEO) [5] platform GPL5345 (n=577). Briefly, positive, unflagged, and non-saturated spots were background-corrected using the median

foreground minus the median background signal intensity for each dye channel and log_2ratios were subsequently calculated from the background-corrected intensities. A probe annotation filter was applied prior to normalization to select only oligonucleotide probes without cross hybridization to other genomic regions or transcripts. Normalization was performed using block-based lowess [8]. After normalization, a probe presence filter was applied to select only probes present in at least 520 of 577 assays (N=10,377), followed by imputation of missing values using weighted nearest neighbor imputation (WENNI). Probes were subsequently merged based on probe ID, and each probe was median-centered across the entire data set.

Molecular subtype classification was performed by correlation to gene expression centroids reported by Hu et al. [4]. Overlapping genes between the 306 genes in the Hu et al. centroids and the 10,377 gene set were identified. Subtypes were assigned to samples based on highest Pearson correlation to a subtype centroid. A correlation cut-off of 0.2 was used as threshold and samples with the highest correlation <0.2 were set as unclassified. Each sample was scored for a proliferation module from Reyal et al. [9] by calculating the average gene expression of genes overlapping between the proliferation module and the 10,377 gene set.

Construction of gene expression centroids based on CGH subtypes and validation in independent gene expression data sets

Gene expression centroids for CGH subtypes were created by first matching the gene list from Hu et al. [4] (~300 genes) to the ~10,300 genes available from the matched gene expression data for the 359 tumors. 203 genes matched and gene expression centroids for

each CGH subtype (Mixed, Amplifier, 17q12, Basal-complex, Luminal-simple, Luminal-complex) were created for these genes by taking the mean for each gene across all samples in a CGH subtype.

A combined Affymetrix U133A data set comprising 1,881 breast tumors from eleven public data sets, including the Chin et al. [3] data set, were prepared and classified according to the Hu et al. molecular subtypes as described [14]. Centroid classification using the CGH subtype gene expression centroids in the Affymetrix data set was performed similarly as for classification using Hu et al. centroids [14] (Pearson correlation, correlation cut-off = 0.2 for unclassified). Unclassified samples were not used in subsequent survival analyses. Survival curves were constructed for luminal A-classified tumors in the combined Affymetrix data set. aCGH data for the Chin et al. data set was used to test whether the luminal-simple / luminal-complex classification of luminal A tumors correlated to difference in FGA in independent data sets. aCGH data for Chin et al. was processed and analyzed as described [17]. The luminal-simple / complex classification of original luminal A tumors in Chin et al. [3] was obtained from the combined Affymetrix data set.
