# Supplementary material for: Genomic subtypes of breast cancer identified by array-comparative genomic hybridization display distinct molecular and clinical characteristics
Source: Breast Cancer Res. 2010 Jun 24;12(3):R42. doi: 10.1186/bcr2596 (PMC2917037; doi:10.1186/bcr2596)
Supplement: Additional file 3 — A pdf file containing one supporting table and six supporting figures. The supporting table describes recurrent high-level amplifications found in the 359 tumors. Supporting Figure 1 describes differences in CNAs and FGA associated with clinical variables in the 359 tumors. Supporting Figure 2 describes differences in CNAs, FGA, and outcome associated with the intrinsic gene-expression subtypes in the 359 tumors. Supporting Figure 3 shows CNA frequency for the intrinsic gene-expression subtypes in the 359 tumors. Supporting Figure 4 describes CNAs associated with the genomic subtypes. Supporting Figure 5 describes differences in CNAs between the luminal-complex and amplifier genomic subtypes. Supporting Figure 6 describes differences and frequencies of CNAs between luminal A tumors classified as luminal-simple or luminal-complex, as well as luminal B tumors classified as luminal-complex. [file bcr2596-S3.pdf]

Supporting Table 1. Recurrent amplifications identified in the 359 tumors

Chromosome	startSRA *	endSRA *	Cytoband	Frequency (%)
1	144052598	145019192	1q21.1	1.1
1	147471646	150705023	1q21.3	1.4
1	154207911	155409658	1q21.3	1.1
1	199898660	204952147	1q32.1-q32.2	2
6	105373057	108147797	6q21	1.4
6	128068280	130405194	6q22.33-q23.1	1
6	134274638	138313123	6q23.2-q23.3	1
8	36836719	38893939	8p12-p11.23	6.7
8	100592699	104688664	8q22.2-q22.3	3.3
8	116278520	119332151	8q23.3-q24.11	6.1
8	124269792	132062486	8q24.13-q24.22	6.4
10	1302776	3832587	10p15.3	1.4
10	7756695	9539483	10p14	1.1
10	78940494	80949616	10q22.3	1.4
11	69132863	70174435	11q13.3-q13.4	5.8
11	76260080	78302333	11q13.5-q14.1	4.2
12	16595	2077521	12p13.33	1.9
12	5508771	7273257	12p13.31	1.7
12	66859847	69360061	12q15	1.7
17	23793112	25006951	17q11.2	3.6
17	34781214	35437549	17q12.1-q21.1	13.1
17	45153711	46560339	17q21.33	3.9
17	51429841	52772356	17q23.2	2.8
17	55207406	57089085	17q23.2	3.3
17	58308724	59993556	17q23.3-q24.1	1.9
19	17275917	19062487	19p13.11	1.1
19	43251702	43876387	19p13.13-p13.2	1.3
19	44919778	45700924	19p13.2	1.3
19	60512441	60850444	19q13.42	1.5
20	54873605	57497912	20q13.31-q13.32	1.3
22	48350551	48884279	22q13.33	1.1

* SRA: shortest region of amplification. Coordinates in HG17 build

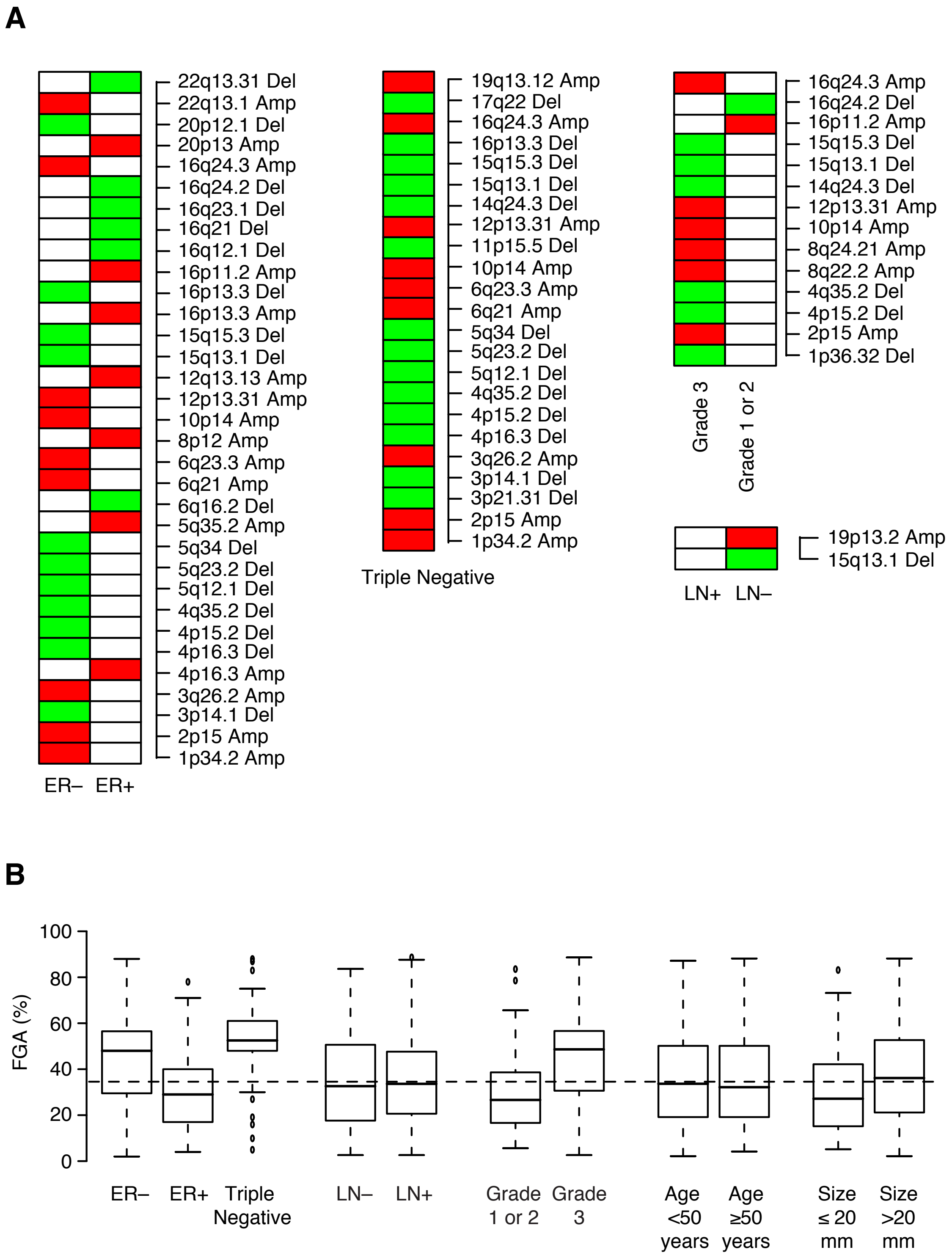

Supporting Figure 1. Differences in CNAs and FGA are associated with clinical variables in the 359 breast cancers. Triple negative tumors are defined as ER-negative, PgR-negative and with average *HER2* copy number < 0.5 in log_2ratio. (**A**) Significant GISTIC regions identified by Bonferroni-adjusted Student's t-test ($p<0.05$) for tumors stratified by different clinical variables. Red indicates more frequent gain in respective group, and green indicates more frequent loss in respective group. Each box represents a GISTIC region. Only significant regions with at least 20% CNA frequency are displayed. (**B**) FGA for tumors stratified by different clinical variables. Horizontal dashed line indicates average FGA for all tumors.

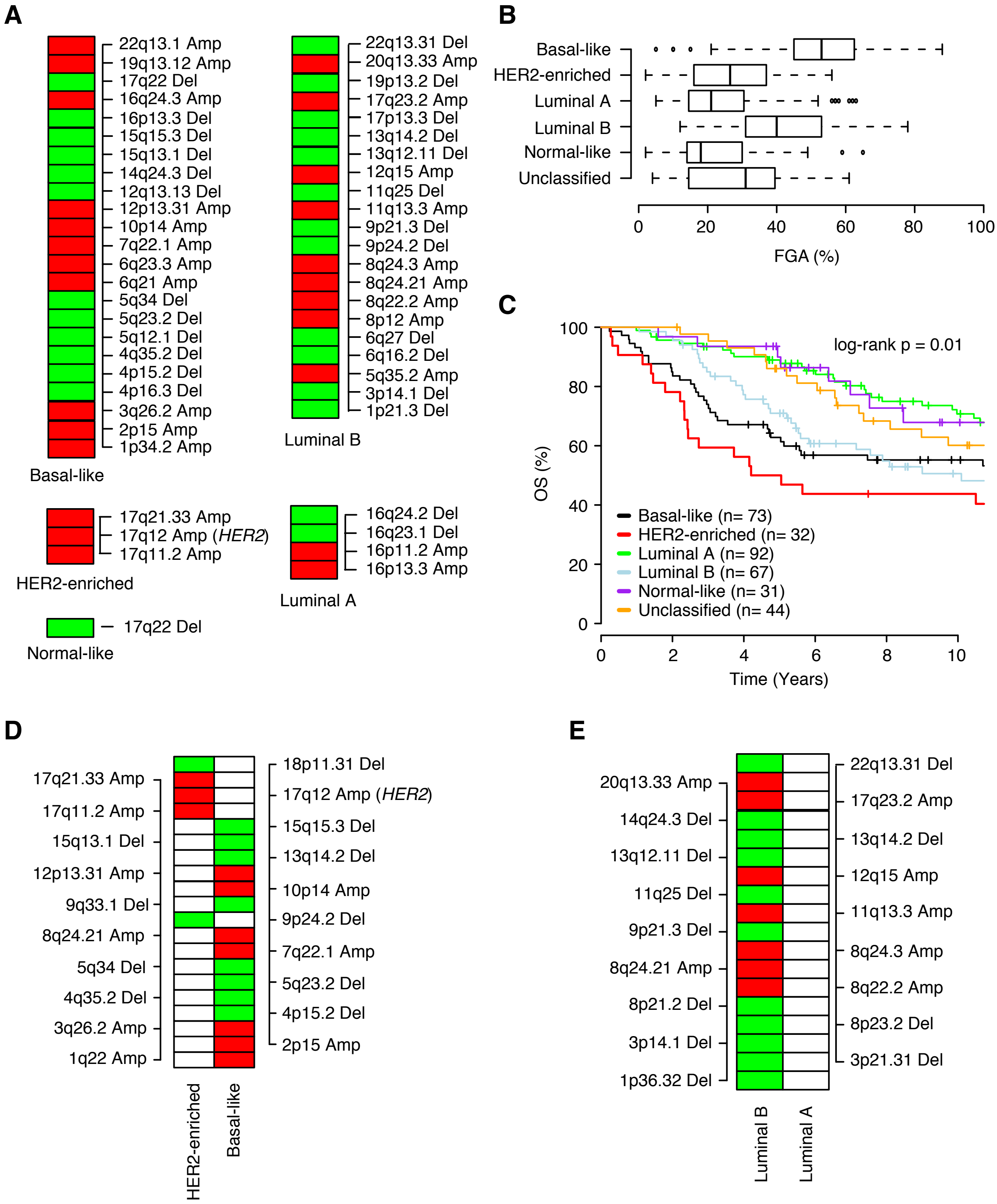

Supporting Figure 2. Differences in CNAs, FGA and outcome are associated with the intrinsic gene expression subtypes of BC. (**A**) Significant GISTIC regions identified by Bonferroni-adjusted Student's t-test ($p<0.05$) for each intrinsic subtype versus all other subtypes combined, excluding unclassified samples. No normal-like specific GISTIC region was identified. Red indicates more frequent gain in respective group, and green indicates more frequent loss in respective group. Each box represents a GISTIC region. Only significant regions with at least 20% CNA frequency are displayed. (**B**) FGA for intrinsic subtypes showing high FGA for basal-like and luminal B tumors. (**C**) Overall survival (OS) for 339 patients where primary tumors were available classified according to intrinsic subtypes. (**D**) Significant GISTIC regions identified by Bonferroni-adjusted Student t-test ($p<0.05$) between the HER2-enriched and basal-like subtypes. Only significant regions with at least 20% CNA frequency are displayed. Several regions were identified, e.g., higher frequency of 17q12 amplifications in the HER2-enriched subtype and more frequent deletions on chromosomes 4q and 5q in basal-like tumors. (**E**) Significant GISTIC regions identified by Bonferroni-adjusted Student's t-test ($p<0.05$) between the luminal A and the luminal B subtypes. Only significant regions with at least 20% CNA frequency are displayed. Several regions were found to be specific for the luminal B subtype when compared to luminal A and included deletions on chromosomes 3p and gains on 8q and 17q.

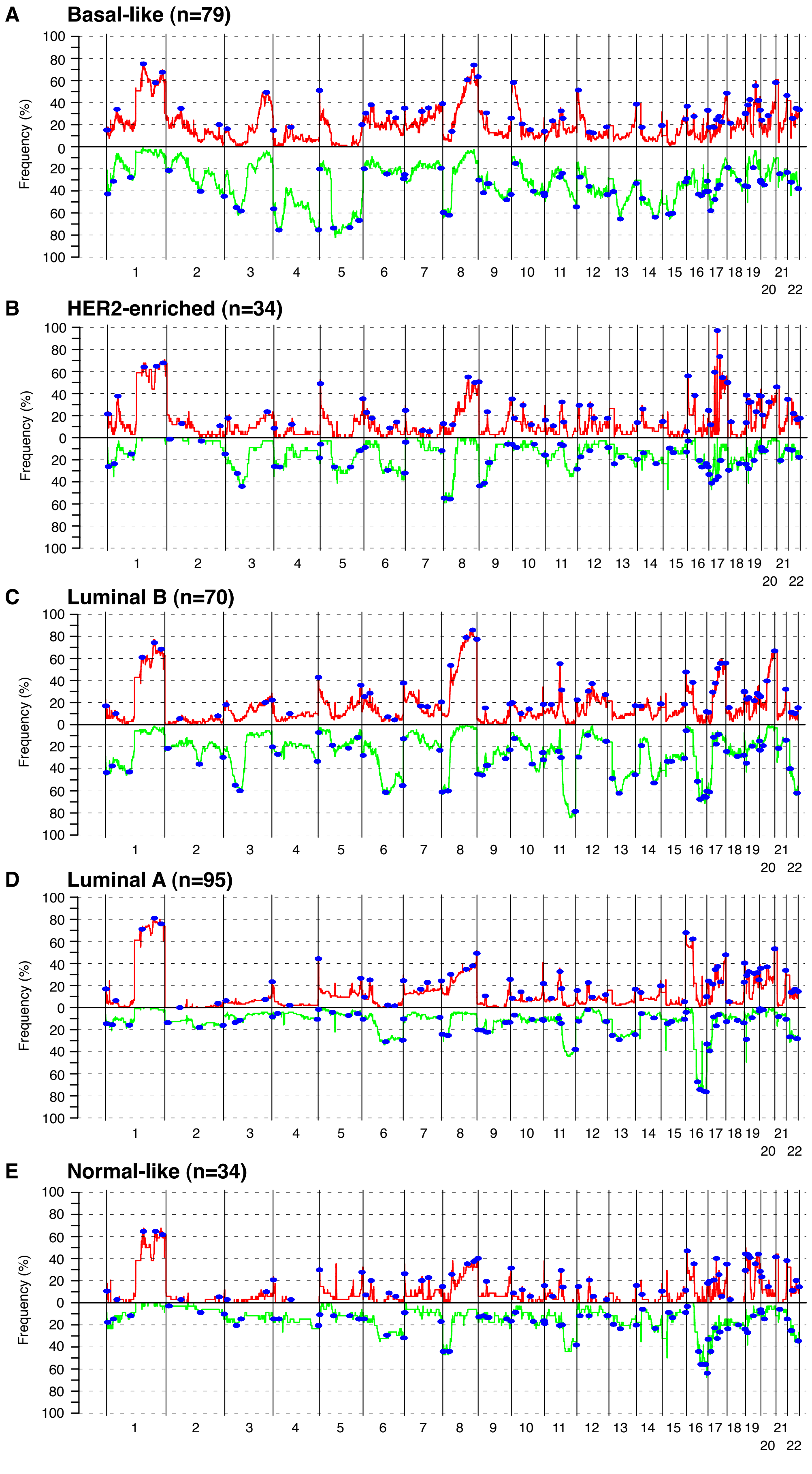

Supporting Figure 3. Frequency of CNAs for 312 breast cancers stratified by classification according to intrinsic gene expression subtypes. Red corresponds to gain, and green to loss. (**A**) 79 tumors classified as basal-like. (**B**) 34 tumors classified as HER2-enriched. (**C**) 70 tumors classified as luminal B. (**D**) 95 tumors classified as luminal A. (**E**) 34 tumors classified as normal-like.

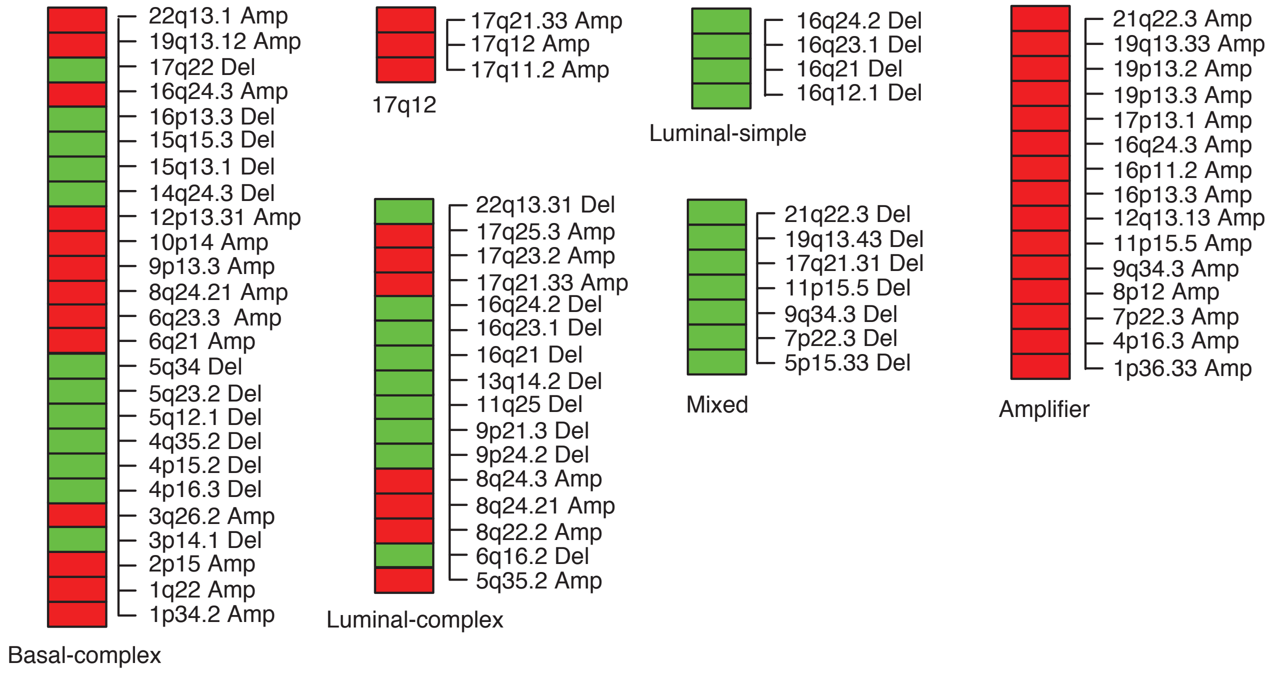

Supporting Figure 4. Supervised analysis identifies specific genomic aberrations associated to individual genomic subtypes. Significant GISTIC regions identified by Bonferroni-adjusted Student's t-test ($p<0.05$) for each genomic subtype versus all other subtypes combined. Red indicates more frequent gain, and green indicates more frequent loss. Each box represents a GISTIC region. Only significant regions with at least 20% CNA frequency are displayed.

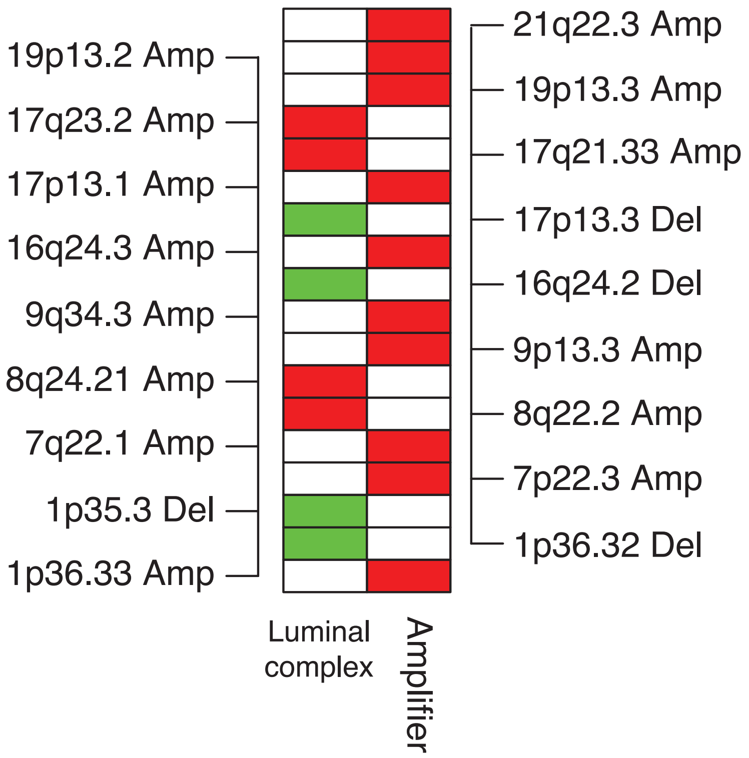

Supporting Figure 5. Supervised analysis between the luminal-complex and amplifier genomic subtypes. Significant GISTIC regions were identified by Bonferroni-adjusted Student's t-test ($p<0.05$), red indicates more frequent gain, and green indicates more frequent loss, in comparisons between GISTIC regions. Only significant regions with at least 20% CNA frequency are displayed.

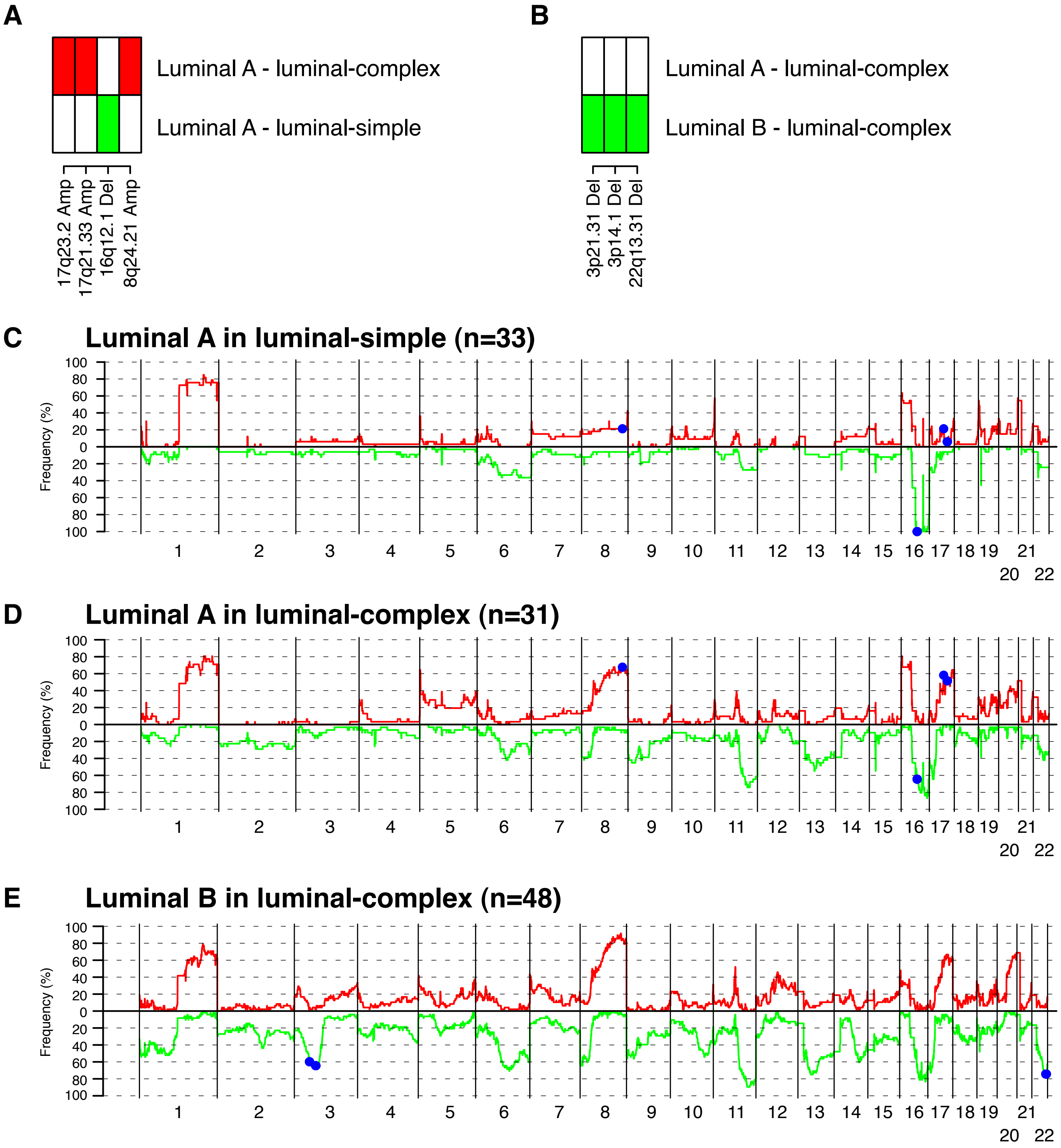

Supporting Figure 6. Supervised analysis identifies CNAs associated with luminal A and B-classified tumors in the luminal-simple and complex genomic subtypes. (**A**) GISTIC regions discriminating luminal A tumors in the luminal-simple vs. luminal-complex subtypes. (**B**) GISTIC regions discriminating luminal A vs. luminal B tumors in the luminal-complex subtype. Significant GISTIC regions identified by Bonferroni-adjusted Student's t-test ($p<0.05$). Red indicates more frequent gain, and green indicates more frequent loss. Each box represents a GISTIC region. Only significant regions with at least 20% CNA frequency are displayed. (**C**) Frequency of CNAs for luminal A-classified tumors in the luminal-simple genomic subtype. Blue regions indicate significant regions from panel A. (**D**) Frequency of CNAs for luminal A-classified tumors in the luminal-complex genomic subtype. Blue regions indicate significant regions from panel A. (**E**) Frequency of CNAs for luminal B-classified tumors in the luminal-complex genomic subtype. Blue regions indicate significant regions from panel B.
