# Supplementary material for: Genomic subtypes of breast cancer identified by array-comparative genomic hybridization display distinct molecular and clinical characteristics
Source: Breast Cancer Res. 2010 Jun 24;12(3):R42. doi: 10.1186/bcr2596 (PMC2917037; doi:10.1186/bcr2596)
Supplement: Additional file 4 — A pdf file showing CNA frequency in the genomic subtypes. [file bcr2596-S4.pdf]

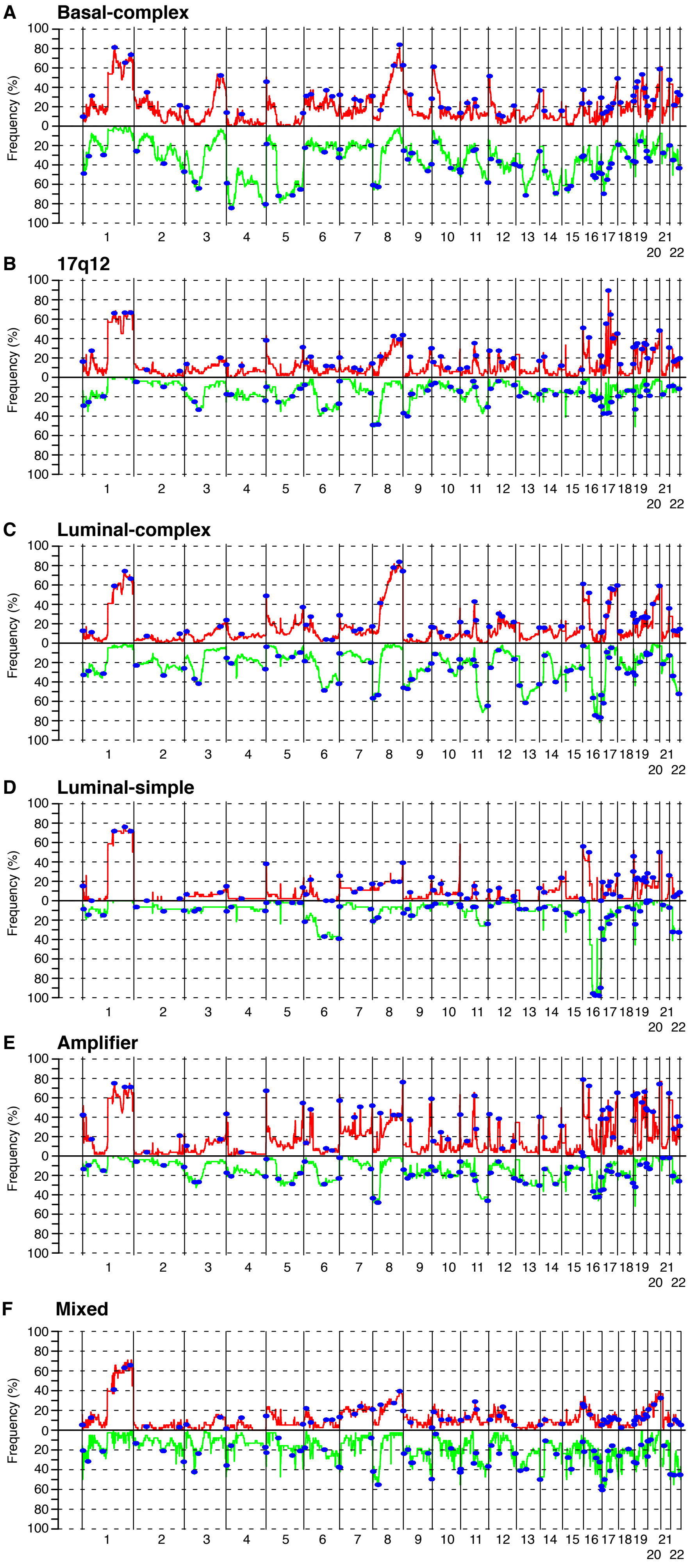

Additional File 4. Frequency of CNAs for all identified genomic subtypes. Red corresponds to gain, and green to loss. Blue regions indicate significant regions (n=133) identified by GISTIC analysis. (**A**) 67 tumors classified as basal-complex. (**B**) 51 tumors classified as 17q12. (**C**) 105 tumors classified as luminal-complex. (**D**) 46 tumors classified as luminal-simple. (**E**) 52 tumors classified as amplifier. (**F**) 38 tumors classified as mixed.
